# Chronic cerebral hypoperfusion induces ABCA1 and apolipoprotein A I mediated cellular cholesterol efflux by activating LXR/RXR in aging rat brain

**DOI:** 10.1186/1750-1326-7-S1-S1

**Published:** 2012-02-07

**Authors:** Linhui Wang, Mingyuan Tian, Li Tang, Bin Liu, Yu Li

**Affiliations:** 1 Department of Pathology, Chongqing Medical University, Chongqing 400016, China; 2 Institute of Neuroscience, Chongqing Medical University, Chongqing 400016, China; 3 Chongqing Key Laboratory of Neurobiology, Chongqing Medical University, Chongqing 400016, China

## Background

Chronic cerebral hypoperfusion has been associated with cognitive decline in aging and Alzheimer’s disease. Vascular dementia produced by permanent, bilateral occlusion of the common carotid arteries in aging rats involves progressive deterioration of intellectual and cognitive function in rats, which are closely associated with the hippocampus. In recent years, it was believed that vascular dementia results from corporation of several factors, in which cholesterol homeostasis and lipoprotein disturbances appear to play an important role. Liver X receptor-β(LXR-β), retinoic X receptor-α(RXR-α), ABCA1 and apolipoprotein A I are thought to be important factors in the mechanism of neurological disease, such as Alzheimer’s disease. But the exact mechanism about vascular dementia is not clear. Here we focus on the mechanism of vascular dementia and offer some new and useful methods in the design of early diagnostic and therapeutic approaches to treat and prevent the progression in vascular dementia.

## Method

Fifty aging male Sprague-Dawley rats aged 12 months and weighing 460~530g, were randomly divided into five groups: a sham-operated group, 1, 2, 3, 4 weeks after 2VO, with 10 rats in each group. The brain tissue lysates were collected for RT-PCR, Western blot assay detecting the expression of LXR-β, RXR-α, ABCA1 and aopA I undering the low blood perfusion. Immunofluorescent double labeled analysis was used to detect the expression and location of LXR-β and RXR-α in the hippocampus in a vascular dementia model. The serum levels of HDL, TC were measured by automatic biochemical analyzer.

## Results

RT-PCR and Western blot results showed that the expression of LXR-β, RXR-α, ABCA1, apoA I mRNA and protein mildly decrease in the 1 weeks after permanent ligation of the bilateral common carotid arteries, compared with a sham-operated group (P<0.05). However, the protein expression peaked at 2 weeks post-surgery (P<0.05), accompanied by severe ischemia. The expression of these proteins decrease at 3 and 4 weeks, accompanied by recovery of cerebral blood flow. Meanwhile, the dynamic alterations of serum levels of HDL and TC were also found (P<0.05). Immunofluorescent staining results not only confirmed the above changes, but also showed that the LXR/RXR heterodimers function as sensors for cellular oxysterols flow in vascular dementia.

## Conclusion

The work highlights the LXR/RXR-ABCA1-apoA I as a protective signaling pathway in vascular dementia. Our study gave further evidence for clarifying the underlying mechanism of Lipid metabolism in a vascular dementia, but more data are needed to firmly establish this protective effect.

